# Multispecies relations shape bird-feeding practices

**DOI:** 10.1038/s44185-025-00080-y

**Published:** 2025-03-18

**Authors:** Tuomas Aivelo, Mikko Aulio, Johanna Enström, Purabi Deshpande, Anna Haukka, Heta Lähdesmäki, Katja Rönkä, Andrea Santangeli, Virpi Väkkärä, Aleksi Lehikoinen, Rose Thorogood, Anttoni Kervinen

**Affiliations:** 1 Science Communication and Society, Faculty of Science, Leiden University, Leiden, the Netherlands; 2 Research Programme in Organismal and Evolutionary Biology, Faculty of Biological and Environmental Sciences, University of Helsinki, Helsinki, Finland; 3 Laurea University of Applied Sciences, Espoo, Finland; 4 Department of Biology, University of Turku, Yliopistonmäki (Vesilinnantie 5), Natura, 20014 Turku, Finland; 5 Helsinki Lab of Ornithology, Finnish Museum of Natural History, University of Helsinki, Helsinki, Finland; 6 University of Turku, Turku, Finland; 7 HiLIFE Helsinki Institute of Life Sciences, University of Helsinki, Helsinki, Finland; 8 Animal Demography and Ecology Unit, Institute for Mediterranean Studies (IMEDEA), CSIC-UIB, 07190 Esporles, Spain; 9 Viikki Teacher Training School, Faculty of Educational Sciences, University of Helsinki, Helsinki, Finland

**Keywords:** Environmental social sciences, Interdisciplinary studies, Biodiversity

## Abstract

While humans often feed birds in their backyards, there is a growing awareness that this has positive and negative effects on local biodiversity. Whether the observed species assemblage shapes human activities has, however, rarely been investigated. We analyzed 15,088 open-ended answers from 9473 Finnish respondents about why they have increased or reduced feeding birds. They mentioned 58 avian and non-avian species linked to changed practices. The main reasons for change were (1) respondent’s relation to nonhuman species, (2) respondent’s relation to other humans, and (3) relations between nonhuman species. Most taxa and reasons could lead to both increase or decrease in feeding, although the direction was context-dependent. We suggest that bird-feeding is an interactive process where the species community strongly affects feeding practices, which in turn can affect community composition. Recognizing this process is crucial for understanding the effects of bird-feeding on both humans and nature and providing more nuanced guidance.

## Introduction

Intended (and unintended) provisioning of wildlife is widespread throughout the world. Across human cultures, people feed animals for different reasons, such as hunting, maintaining ecosystem services or performing scientific studies, but it is especially common as a hobby^1^. Indeed, bird-feeding might be the most common interaction between humans and wild animals in the Global North^2^ and constitutes one of the most common and consequential forms of care that humans give towards other species. For example, in the UK, three times more feed intended for birds is sold annually than the populations of the ten most common garden birds can theoretically consume^3^. While feeding birds brings benefits to people, there is nevertheless growing concern that bird-feeding practices may have unintended consequences on nature^4^. Although providing food enables some species to better survive the winter or increase breeding productivity, it can also shape synanthropic birds^5,6^ and non-avian populations^7^. This practice also has wide-ranging effects on ecological communities through the spread of pathogens^8^, competition^9^ or predation^10,11^. All in all, it is still unclear whether the overall effect of bird-feeding on biodiversity is net positive or negative^3,12^. As bird-feeding is a culturally and socially varying practice (such as when, where, by whom, and how birds are fed)^6^, if we are to understand how bird-feeding affects ecological cascades and biodiversity, it will be crucial to elucidate the drivers behind changes in these human practices.

The essential driver of bird-feeding is simply that people want, for various reasons, to feed birds^6,12^. In multiple studies of their motivations, people report that feeding esthetically pleasing species, or caring for species perceived as in need, brings pleasure and enhances their own wellbeing^2,13,14^. This may come from a desire to nurture nature, to enhance opportunities to observe and educate themselves about different species, to provide a sense of companionship, or even to make amends for apparent negative effects on nature^13^. Bird-feeding is also increasingly framed as a tool to “(re)connect” people with nature^15,16^, echoing the pervasive Western human-nature divide^17^. However, and perhaps due to this emphasis on benefits to people, there are still relatively few studies on how the wanted or unwanted species that are observed at bird-feeding sites affect humans’ bird-feeding practices^1,6,12^.

Bird-feeding has a broad nonhuman component that could influence decisions of what, where, and when to feed. In one of the few studies investigating how observations of nature influence feeding activities, Dayer et al.^12^ found that a scenario of observing a predator such as a domestic cat was coupled with a range of hypothetical actions: for example, respondents would scare off the cat, improve the shelter available for birds, and relocate feeders to make them less accessible to cats. People may also adjust their behavior differently depending on variations in how they value the species they observe, as there is ample evidence that people value different species in different ways^18^. Dayer et al.^12^ found that a scenario of observing a hawk at feeders led some respondents to suggest similar changes as in relation to the presence of cats, but in contrast, half of the respondents stated that a hawk would not change any of their feeding practices due to it being a “natural predator” and part of the “circle of life”. Indeed, even perceived interactions with “undesirable” non-target species can potentially have large impacts on changes in bird-feeding actions: in a recent study using part of the dataset that we use here^19^, the risk of rats being attracted to the site was the most common reason given for why people had reduced their bird-feeding activities—and this seemed to be encouraged more by rules and regulations rather than direct observation of rats^20^. Bird-feeding can, therefore, be considered an interesting case of human value judgments and trade-offs between competing interests. This means that it would be better studied as a continuous multispecies interaction where humans offer food, nonhuman species either take up or do not take up the offer, and humans adjust their feeding practices accordingly^20^. While previous studies used predetermined lists of species and scenarios, by exploring respondents’ descriptions of situations that have led in real life to decrease or increase bird-feeding, here we can dig deeper into the species that are described as wanted or unwanted, and in the human behavior in relation to biodiversity.

In this study, we therefore focus on multispecies relations to investigate the drivers that lead to changes in bird-feeding practices. There is ample time for people’s preferences and multispecies interactions to manifest, as bird-feeding is often a long-time commitment. People choose to feed birds for years^19^, and adjustments to feeding practices are more often in response to observations of nature than to human-centered constraints of time and money^12^. However, as scholarship on multispecies care has shown us^21^, the care provided through bird-feeding is likely to be far from “innocent”^21^, with potential behavioral and even morphological changes in the birds utilizing the food provided^22–24^. We suggest that zooming into individual events and species appearing at the bird feeder and then following reciprocal (re)actions should enable us to better understand humans’ relations to nonhuman species and vice versa. We expect that any changes in feeding practices will expose important motivations, conflicts, and trade-offs that people experience in between-species relations.

We utilize a large dataset of self-reported responses to an online questionnaire where 9473 residents of Finland described events that have influenced changes in their bird-feeding practices over the last 20 years. As these changes relate to the actual events and their social and ecological consequences, we can delve deeper than inquiring about human behavior in hypothetical situations. Bird-feeding has a long history in Finland, spanning from the 19th century^20^, and currently, bird-feeding is a widespread hobby across the country^19^. Nevertheless, the number of people feeding birds is diminishing although the amount of food being provisioned has increased in some cases^19^. At the same time, bird communities are rapidly changing due to, e.g., climate change^25,26^ with implications for broader ecological processes, particularly in urban environments^27^. Therefore, bird-feeding in Finland provides an ideal context to study human-animal interplay and multispecies relations. We asked (1) what explains the changes in people’s bird-feeding practices, and specifically, (2) which species affect bird-feeding practices. We expect that people will report reducing feeding activities when species perceived as unwanted appear at the feeding site, or the presence of wanted species will have led to increases in bird-feeding, with value-based statements thus revealing variation in attitudes towards different species.

## Results

### Reasons for changing feeding

Of the 9473 survey respondents, 4992 responses included a reason for making changes in bird-feeding. We categorized these as follows, (1) the respondent’s relation to another species, (2) the respondent’s relation to other humans, and (3) relations between other species. These were then divided into seven subcategories, containing 26 individual reasons (Table 1). Of the main categories, “Respondent’s relation to another species” contained 86% of all classified responses. Most (67%) were then classified into the subcategory regarding “Attitudes and interest towards birds and nature”, and within this, the most numerous and second most numerous reasons given were in regard to feeding certain species and not others (Supporting wanted species, 38%/Lack of wanted birds or presence of unwanted birds 16%). By comparison, very few reasons were given that could be categorized as referring to relationships with other people or among other species (i.e., “Respondent’s relation to humans” and “Relations among nonhuman species”; in contrast, other people and relations between other species both comprised 7% of reasons).

**Table 1:** The main categories, subcategories and individual reasons for increasing or decreasing bird-feeding

Main category	Subcategory	*n*	Reason for increasing feeding	*n*	Example	Reason for decreasing feeding	*n*	Example
Respondent’s relation to nonhuman species	Attitude and interest towards nature or birds	3328	Empathy, pity	248	“I noticed that the birds are very hungry when I gave them nuts from my hand.”	Empathy, pity	10	“I do not think it is sensible to tinker with birds’ habits and their probabilities of surviving the winter.”
			Interest in birds or nature	306	“I got interested in bird species identification.”	Interest in birds or nature	1	“I moved the feeder to the shade so that it is easier to identify birds.”
			Healthier or tastier food to birds	61	“I’ve changed the feed that I provide based on what birds eat.”	Picky birds	17	“Suet balls weren’t eaten.”
			Supporting wanted species	793	“Because there’s also other species than birds around, such as rabbits, deer, raccoon dogs and squirrels.”	Lack of wanted or presence of unwanted species	1892	“Too many sparrows on the feeder, only two wanted species (great and blue tit) on the feeder.”
	Diseases	941				Zoonosis	520	“I have plants on the balcony and I don’t want the birds to touch them as I’m worried about pathogens spreading from bird feces.”
						Pathogen spread between birds	421	“There were several dead birds two winters ago.”
	Economic reasons	28				Too much demand for food	28	“Jackdaws came to the feeder meant for the small birds. No food left for small birds and upkeep was expensive.”
	Scientific reasons	6	Doing research	5	“I do research on the overwintering of birds that use feeders.”	Stopping doing research	1	“I used to ring birds in the winter.”
Respondent’s relation to humans	Social relations	169	Counterreaction to social pressure	1	“People are afraid of rats and blame people feeding birds. It just increases my interest in feeding birds and squirrels.”	Social pressure	91	“My spouse prohibited [feeding] because of the rats.”
						Sabotage	20	“Neighbors are bothered, suet ball holders are stolen and [feeding] trees are being watched. All feeders are destroyed in the area.”
			Lack of human contact	1	“It is nice to observe birds especially due to social distancing (covid).”			
			Traditions	30	“I feel it is important to teach children how wildlife can be taken care of.”	Children moving away	11	“Children have moved away. It was nice to observe birds with them.”
			Others are feeding	1	“I dared to start feeding as I saw my neighbor doing it.”	Others are feeding	14	“Somewhere close by, there’s better selection and birds are rarely in my yard. I do not want to get into an arms race.”
	Rules	187				Prohibitions	187	“The housing association has prohibited feeding due to an increase in the rat population.”
Relations among nonhuman species	Ecological reasoning	333	Supporting biodiversity	143	“We want to support biodiversity around our cottage.”	Effects on bird community	69	“Feeding changes the bird community too much.”
						Unnecessary	22	“I feed only from December to March as the birds do not need it otherwise.”
			Pets, domestic animals or garden	24	“Functions as a television for our indoor cats.”	Pets, domestic animals or garden	75	“Tits come to the feeder and then they go to the barn to peck on cows’ udders.”

The number of responses is shown on the level of subcategories and individual reasons. If the same reason can lead to both decrease and increase in bird-feeding, those are placed on the same row in the table. The responses were given in Finnish or Swedish and translated here to English for presentation.

Among the main categories of responses, those associated with the respondent’s relation to humans led more often to negative changes in feeding (33 positive changes to 323 negative, binomial test = 0.90, *p* < 0.001). While the relation to nonhuman species was more evenly spread (1413 positive changes to 2890 negative; Table 1), these reasons were still more likely to lead to a decrease in providing food for birds (binomial test = 0.67, *p* < 0.001). On the other hand, reasons associated with relations among nonhuman species were as likely to produce positive or negative outcomes on feeding behavior (167 positive changes to 166 negative, binomial test = 0.50, *p* = 1). This may reflect respondents being more likely to complete the survey if they had reduced their feeding (see ref. ^19^). There were also interesting differences in reasons given that led to the same outcome. For example, “Supporting wanted species” was the reason that occurred most often for increasing feeding (“Relation to nonhuman species”), whereas zoonosis and pathogen spread between birds (“Relation to nonhuman species”), sabotage and (housing) rules (“Relations with humans”) were the reasons that led only to a reduction in feeding (Table 1). Many reasons also formed pairs, where the same event and reasoning could lead to different consequences on the feeding practices. For example, events at the bird feeder itself, such as squirrels or magpies eating a lot of food, could lead to either the person providing a lot more feed, or completely stopping feeding; or empathy for birds could encourage feeding as well as the cessation of the activity:

“I pity birds during the freezing weather.”

- Respondent 10706

“[Birds] could have gotten used to feeding and, when there would have not been supplemental food, they would have suffered.”

- Respondent 10

Nevertheless, these pairs were clearly more often negative or positive-oriented (Table 1). For example, empathy and pity led mostly to increased feeding (248 to 10) whereas social pressure led to a decrease (1 to 91).

Ecological thinking also came into play to create more complex scenarios such as:

“Too many mice, voles at the feeding site, leads to increase in snakes in the backyard.”

- Respondent 8907

It was unclear from answers like these, however whether a change in feeding behavior was a result of a perceived or actual indirect effect on biodiversity (i.e., feeding birds > rodent numbers > presence of snakes). Additionally, some respondents were worried that birds of prey would eat smaller birds attracted to the feeders (*n* = 6), whereas others found this as a reason to feed small birds if it could attract birds of prey to their backyard (*n* = 2). This showed strong evidence that people can also place different values on species that are generally considered as wanted.

### Species and species groups that affect feeding practices

**Fig. 1:**
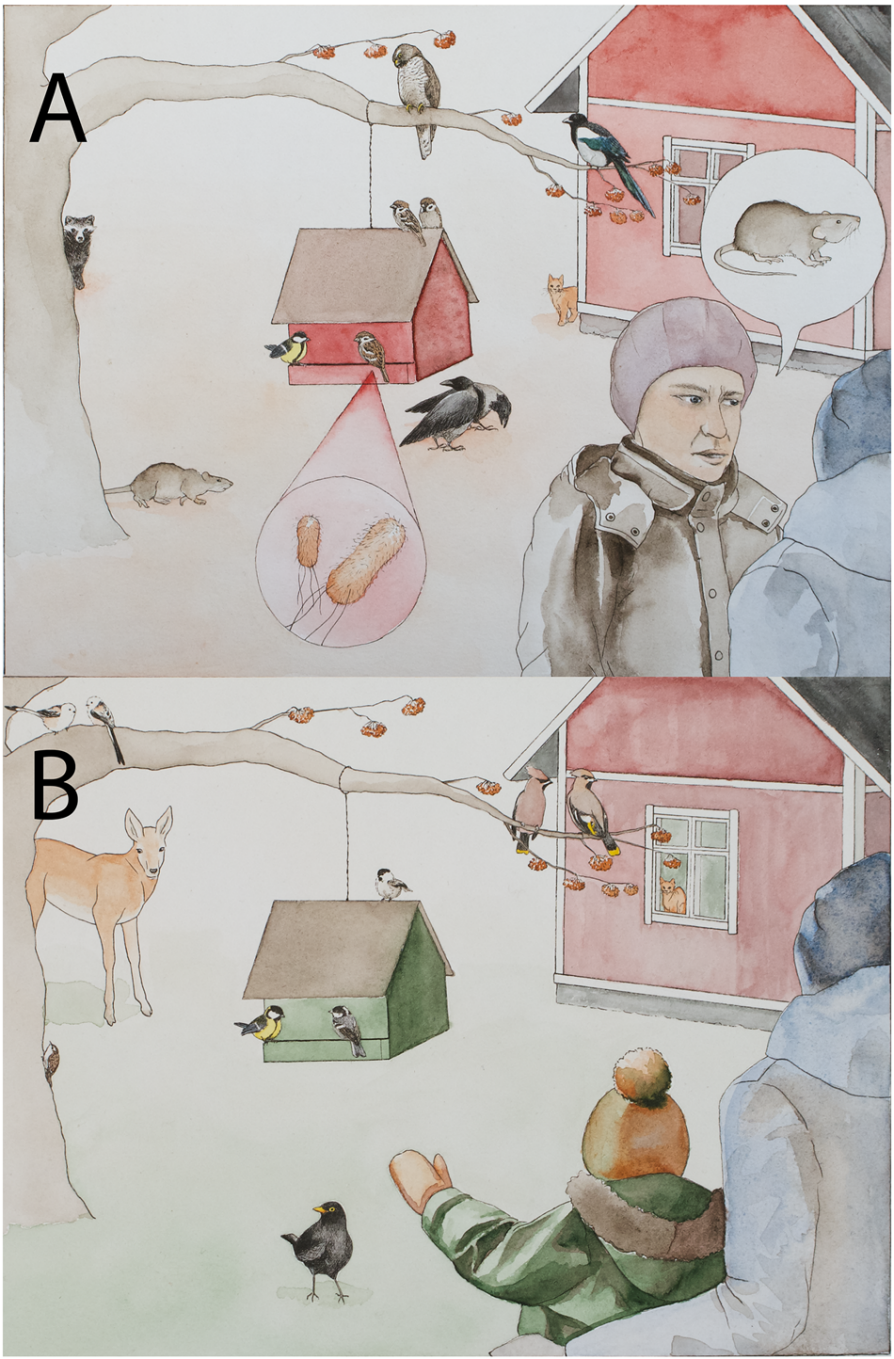
Illustration of reasons to change bird-feeding practices. Species and contexts that lead to (**A**) a decrease in feeding birds and to (**B**) an increase in feeding birds. Drawn by Susu Rytteri.

A total of 1814 mentions of 58 different species or species groups were named as primary reasons for changing feeding practices (Table 2 and Fig. 1). Most of the species that affected bird-feeding habits were animals, and birds and mammals had the most mentions (85% of mentions; 49 out of 58 taxa). These seem to reflect the wide diversity of different feeders and feeding methods that people use, not only targeting small birds, but also larger species.

**Table 2:** Classification of mentioned taxa within higher-level taxa

Organismal group	Examples	Decrease	Increase
Pathogens	Bird flu, salmonella	111	
Ectoparasites	Ticks, fleas	7	
Waterfowl	Swans, ducks		8
Birds of prey	Owls and accipitrids	6	2
Other large-sized birds	Pigeons, seagulls, corvids	187	46
Woodpeckers	Black woodpecker, great spotted woodpecker	11	8
Other medium-sized birds	Thrushes, Bohemian waxwing		20
Tits	Great tit, blue tit	10	18
Other small birds	Sparrows, yellowhammer	4	26
Pets	Dog, cat	40	36
Wild carnivores	Raccoon dog, red fox, mustelids	12	3
Deer	White-tailed deer, roe deer	7	13
Hares	Hares, rabbit	18	21
Red squirrel		99	67
Other rodents	Brown rat, mice, voles	1005	5
Shrews		4	
Snakes		1	

For each taxon, the columns show the number of mentions related to either decreased or increased feeding. Taxa are grouped to contrast the responses, wherever interesting; for example, woodpeckers more often led to decreased than increased feeding, whereas other medium-sized birds were without exception linked to increased feeding.

The most mentioned taxa overall, as well as in only the answers linked to a negative change in feeding, were rats (586 mentions of decreased feeding, 1 of increased), followed by mice (269 decreased, 3 increased), corvids (156 decreased, 34 increased), and pathogens (111 decreased). Mammals were markedly related to decreased feeding (though not always), and they could even lead to stopping feeding altogether. In contrast, the species that led to increased feeding were spread more evenly across taxa. Waterfowl, thrushes, and other medium-sized birds, such as waxwings, were the only species mentioned that caused an increase, but not a decrease in decisions to provide food to birds.

Most of the mentioned taxa were linked to both an increase and decrease in feeding. For example, cats affected bird-feeding negatively when it was the neighbors’ cats entering the feeding site and attacking birds, whereas a respondent’s own cat could lead an increase if feeding provided entertainment for the cats, e.g., by following the activities at the bird feeder safely through a window. It should be noted that increases or decreases in feeding do not necessarily relate to a person’s affective perception towards the species. For example, a species can eat so much food, that it requires increased food provision to make sure that other species have enough. This, in turn, could either lead to an actual increase in feeding or it could lead to a decrease if, for example, the person could not afford to buy more food and, therefore, decided to stop feeding altogether.

## Discussion

While the focus of bird-feeding studies has been either on the effects that bird-feeding has on avian or non-avian populations and communities^9,28–30^ or the reasons why humans want to feed birds^2,6,31–33^, our approach reveals a more complex diversity of ways in how birds and other species attracted to bird-feeding sites can, in turn, affect how people provide food for birds. The species present or absent at the bird feeder can both affect the bird-feeding practices, but, interestingly, most of the species can, depending on the context, lead to both increases or decreases in feeding birds. Context is highly important for many taxa: for example, cats are often seen as unwanted when a neighbor’s cat is predating birds at a feeding site, but when the owner’s cats are inside and watching birds through the window, they can provide a reason to feed birds. Sometimes, making decisions based on multispecies relations may lead to potential trade-offs, such as a respondent who said that there is no problem with the seeds that fall to the ground and attract rodents as free-roaming cats kill those rodents. The wild predators were less common than cats, but they predominantly led to a reduction in feeding birds. Interestingly, this provides potential for conflicting responses as the small birds on bird feeders attract predators^34^, though some respondents also felt happy about seeing birds of prey at their feeders. Indeed, our approach of asking about the changes in the feeding practices revealed a plethora of primary reasons that interact with species when people are making decisions on whether to put more or less effort into bird-feeding. Thus, clearly, bird-feeding is not a one-way interaction, but rather a series of reciprocal relations where humans and nonhumans react to each other.

Our results show that the most common species that leads to a decrease in bird-feeding in Finland is the brown rat. The most important reason to increase feeding was the occurrence of wanted species in the bird feeders. These are not surprising results as they correspond well to previous findings^1,12,19,35,36^. Interestingly, the most often mentioned taxa and most often mentioned reasons were both related to the decrease in bird-feeding rather than those that people were aiming to attract. This significance of unwanted species is also manifested in the apparatus used to provide food as avoiding feeding unwanted species drives the evolution of the bird feeder models used, more than improvements for feeding the species people prefer^20^.

Closer inspection also suggests that there are more complex phenomena taking place across bird-feeding sites: the same taxa can lead to an increase or decrease in feeding, but the same reason in different contexts can lead to either an increase or a decrease in feeding. The red squirrel (*Sciurus vulgaris*) is an interesting example, as it can be both a wanted or unwanted species at the bird feeder. Regardless of its status, squirrel appearance could lead to an increase in provisioning to make sure that birds also get food, or it could lead to feeding birds being stopped altogether due to concern that the squirrel may eat too more food than the respondent was prepared to invest in bird-feeding. Indeed, our results corroborate and expand on Dayer et al.’s^12^ findings that there can be both positive and negative feedback loops between observations of species at the bird feeders, human attitudes, and actions, and subsequently changes in the species community at feeding sites. Similarly, the disappearance of a wanted species from the feeding site can lead to both an increase and decrease in feeding birds. The respondents described ending the feeding completely, increasing the amount of feed or changing feed qualitatively in response to the situation. While some reasons can be expected to lead to a certain direction of change (for example, pathogens always lead to decreasing feeding), there is still a variation in whether the change is cessation, reduction, moving the feeding site or changing the food provided.

Why might people vary so much in how they relate to nonhuman species and whether their perceptions lead to increases or decreases in feeding birds? One possible reason is that our survey data was not obtained from a geographically large area across urban and rural areas. Indeed, in previous work, we found that bird-feeding occurs more often in urban areas, but is declining, while more food is provided in rural areas where it is increasing^19^. In other countries such as Poland, there can be large differences in the number and type of feeders provided, as well as the species that are attracted to them^37^. This could, therefore, influence the feedback loops that we have identified here between the species present at feeders and people’s relations to them. Indeed, the values placed on wildlife, as well as feeding birds, can vary widely depending on urbanity, socioeconomic status, household size, and age^38^, so an important next step will be to tease apart whether the feedback loops we describe here might also vary and potentially contribute to emerging differences in, e.g., urban-rural bird communities^29^.

A second reason is that people vary in how they appreciate or rate the “attractiveness” of species, and this can sometimes be acknowledged directly or indirectly. For example, the long-tailed tit (*Aegithalos caudatus*) was considered to be cute, and thus, they were fed. In contrast, some people explained that they attract birds and ensure their survival by reducing the pest insect pressure for the garden in the summer. These findings are not surprising as they resonate with previous research: for example, the long-tailed tit is esthetically pleasing to people^39^. On the other hand, some species, such as rats, are a typical example of both direct and indirect reasonings as they can be unwanted based on people just not liking them^40,41^, but also rationalized through reasons of zoonotic or infrastructure risks (even if the actual effect could be minimal or mitigated^42,43^). Indeed, these perceived risks can lead to regulations and prohibitions, which might lead to stopping feeding, even though the person feeding would have liked to continue (although our questionnaire exposed that regulations or social pressure are not always acted upon, see below). Many respondents were also worried about the ecological effects of feeding birds. If bird-feeding has been initially seen as targeted at improving the welfare of individual birds^20^, nowadays, respondents were clearly also thinking about the effects on specific, usually locally threatened species, and to biodiversity at large. This can be seen, for example, in the responses where observing tits at the feeding site could reduce feeding. There has been discussion about synanthropic tits (Eurasian blue tit, *Cyaniste caeruleus*; great tit, *Parus major*) benefitting at the expense of non-synanthropic tit species (willow tit, *Poecile montanus*; coal tit, *Periparus ater*): as their survival increases during the wintertime bird-feeding, they can dominate the community during the breeding season^44,45^. This knowledge has led to some respondents reporting being worried about willow tits as a reason to stop bird-feeding altogether, although recent results from Finland do not support this interaction among tit species^46^. Thus, different perceptions of the desirability of species and context could lead to different outcomes when it comes to feeding birds.

Third, our results highlight that interpersonal relations among people can also influence how they interact with nonhuman animals. For example, social conventions, relations and norms affected respondents’ decisions of whether to change how they feed birds. These norms could be explicit, either as written rules or neighbors shouting over the fence, or implicit, such as perceived acceptability in the neighborhood^31^. In Finland, urban apartment buildings are commonly administered as housing associations, where every apartment owner has a vote, and these associations commonly prohibit bird-feeding in the common yard due to, e.g., concerns over the presence of rats^47^. Importantly, however, we found that these norms did not always lead to consistent change, with some respondents even finding social pressures to be an inspiration to increase or even start to feed birds. Nevertheless, the most common relation that affected bird-feeding among our respondents was human-to-other species relation, and this was expected, as bird-feeding is generally expected to function on humans’ terms and conditions. For example, magpie can be seen as “stealing” food from the bird feeder: this might be due to people not wanting to feed magpies as they want to feed rather small birds or also because magpies, as large-sized birds, eat much more and while people might not have anything against magpies as such, they cannot afford feeding magpies. Some similar reasoning could be also seen through how people care: many people care for nonhuman animal-to-animal relations, such as evidenced by reasons for feeding birds being to provide entertainment for one’s pets. It seems that cultural norms, such as small birds being in more apparent need of care than corvids, are in play here.

Our findings should be understood within the Finnish context. For example, in Finland bird-feeding has long been more common during the winter, in particular when there is snow on the ground. Feeding birds when there is no snow on the ground is much less common and is more often directed to waterfowl or to birds in urban parks^19,48^. The total number of respondents to our questionnaire (i.e., more than 9000) was close to 0.5 percent of all households in Finland (i.e., 2,793,636 in 2021)^49^. About half of the households in the UK feed birds^1^, and although this data is not available for Finland, it seems that feeding birds is likely to be as popular here. For example, the amount of food (per household) imported to both countries intended for feeding wild birds is similar (UK: 28.4 million households purchase 150 million kg^50^; Finland: 2.7 million households with 15.8 million kg imported^51^). As the answers to our open questions were informative, even if sometimes short, such as “My heart was so broken when the magpie found the suet balls,” we would expect that our qualitative data should contain any important phenomena that would occur. We suggest, therefore, that the underlying phenomena we have found in this study might be quite universal, such as is shown by the global surge in bird-feeding during the pandemic^52^. On the nonhuman animal side, even if the particular species are different in different locations, their ecological niches vary similarly to Finland and are thus likely to present similar observations and interactions in bird-feeding sites. Thus, we suggest that while the specific species and proportions of different reasons vary from country to country, these relations are likely to be present in some form across the globe. More broadly, entering into these relations through bird-feeding creates a cycle of action and reaction: for example, feeding birds lead to behavioral changes in birds that not only modulates the pleasure that humans are able to derive from observing birds, but also relate to interactions among the bird community^22,23^.

Asking about the changes in bird-feeding practices and their underlying reasons directly links human actions and the relevant observations that humans make in their bird feeders. This approach does depend on the human perception about the change in the feeding. We do not know about the changes that are perceived as neutral, nor can we be sure whether the humans’ perception of the direction of the change is the real effect on birds. For example, a study showed that while using a squirrel guard increased the amount of feed going to birds, it reduced bird visits to the bird feeder and had differential effects on different bird species^32^. Similarly, we are not able to assess how relevant different perceptions of diseases are and whether they correlate with actual risk to humans or birds: while they are a highly common reason to reduce feeding, there are already a number of ways of taking these into account, such as bird-feeder design^20^.

In conclusion, we suggest that the relationship between bird-feeding and humans’ animal attitudes is a highly dynamic process and also context-dependent: the same species can in different contexts lead to either increase or decrease of provisional feeding. Similarly, the same reasons can be used to justify increases and decreases in feeding. Thus, the avian and non-avian species that are directly or indirectly affected by bird-feeding also affect humans and become actionable participants in the process. This phenomenon has both ecological and social dimensions, which are tightly interlinked. While bird-feeding influences species communities, it is also important to acknowledge and understand how other species influence feeding practices and behaviors and are active participants in bird-feeding practices.

## Methods

### Questionnaire design and participation

We designed a questionnaire to better understand how and why people feed birds in Finland^19^. The questionnaire was prepared in Finnish and Swedish (the official majority languages of Finland) using the Google Forms platform and was available for two months between January 29 and March 31, 2021. Participation was solicited openly through the social media channels of the Finnish Museum of Natural History Luomus, through the local chapters of BirdLife Finland, and by publicity through television interviews and online news provided by the Finnish Broadcasting Company YLE; the questionnaire could be accessed through the website of the Finnish Museum of Natural History Luomus or by direct link.

While the questionnaire included 13 questions^19^, only the four questions with open-ended answers are analysed in detail here. It is noteworthy that the questionnaire did not limit respondents to answering about bird-feeding to, e.g., only during the winter or to feeding done in the respondents’ own yard, though these were clearly emphasized in the answers. The open-ended questions were (translated): “Have you changed your bird-feeding habits within the last ~20 years?”; “Why have you changed your feeding patterns?”; “If you have stopped feeding birds did any or several of the following factors influence the decision to quit?”; and “Give feedback to the creators of the questionnaire.” The first three of these questions had closed options, but also an open-ended alternative. As similar answers could be given to more than one open-ended question, all answers by a single respondent have been analyzed together. The respondents that gave discrepant answers between closed and open alternatives (e.g., they had answered in the closed option that they did not change feeding, but then explained in the open alternative answer the ways that they had changed feeding) were removed from further analysis.

### Qualitative analysis

We used data-driven content analysis to categorize individual responses through coding, i.e., “looking for pattern or order in a body of data by identifying recurring themes, categories or concepts”^53^. The unit of analysis was either a full or partial answer which contained a comprehensible thought and these varied from single words to several sentences. First, we coded the reasons for the change in bird-feeding whether this was a species or some other reason. Secondly, the units of analysis were coded based on the direction of the change: decrease or increase. Every applicable reason for the change was coded as either positive or negative. The open-ended questions either asked for (a) the reasons behind any type of change (here the direction was inferred unless explicitly specified by the respondent), or (b) the reasons behind stopping feeding altogether (here the direction was explicit). *Decrease* contained decrease in the amount of feed or the span of feeding time, moving the feeding site due to human-centric reasons (i.e., the feeding site was changed to make feeding easier for humans) or changing the feed to cheaper, whereas increase could be increase in the feed or the span of feeding time, moving the feeding site due to bird-related reasons (i.e., moving to a place with more birds or to safer location) and changing feed to a sort perceived more healthy or tastier. Coding was done in the qualitative data analysis software ATLAS.ti (v. 23.3.0 Mac; ATLAS.ti Scientific Software Development GmbH, Berlin, Germany). All coding and categorizations were completed using the original language of the respondent and translated into English (with the assistance of native English speakers) for discussion with co-authors and presentation purposes. Although coding and interpretation was qualitative, we supported numerical comparisons where possible using binomial tests, implemented in R version 4.4.2^54^.

Initial coding by JE generated a rich and diverse set of 834 codes to describe the whole phenomenon. Then the coding was focused by MA to account for all instances when a species was indicated as a reason for changing feeding practices. At this point, about 1000 responses (out of 9473) were cross-checked to assess the initial coding: the taxon- and direction-specific codes were deemed suitable for the purposes of this study, and there were no mismatches between existing taxon names and responses, but these codes were used as is, i.e., inter-coder reliability was 100%. Additional species that were missing from the coding (as they were, e.g., originally coded on a higher taxonomic level) were added at this point, increasing the set to 954 codes. Initial codes also included several answers which contained the respondents’ thoughts on why other people have changed their feeding practices, and these were removed. In addition, some codes were removed due to a clear conflict between a respondent’s closed and open-ended answers (e.g., claiming to have increased feeding while explicitly mentioning only having decreased feeding) or deemed not relevant to the research questions. Thus, the final set included 216 codes which were further classified thematically to create different categories of reasons and to include their directions of change.

The initial codes contained sometimes long causal chains, such as nearby forest was clear-cut which led to decrease in the number of birds in the feeding site. In such cases, only the most proximal reason and taxon (here: lack of wanted birds) were used. Additionally, humans, the coronavirus, pet chickens and species offered as a feed, such as pork (lard) or peanut, were removed at this point.

The categorization of the reasons for the change in feeding was challenging as the number of codes was substantial, and codes could create hierarchical relationships [e.g., (1) Corvids as (2) big birds spread food around, which (3) makes a mess, which in turn attracts (4) rats, which come (5) inside the building and do (6) damage]. The codes were, therefore, then organized and classified in visual networks. These networks were first built with the core codes that contained many responses, then organized into main categories, and then further split into subcategories. Finally, the remaining codes were added one by one to the network. Each code belonged to only one subcategory, but the subcategories could include codes with opposite directions of change.

It is notable that on a main category level, both humans and nonhumans can be present in any category. For example, the relations between humans are facilitated by complex chains of events:

“My neighbors did not like the hulls of sunflower seeds on their terrace, so I changed to hulled seeds.”

- Respondent 516

The primary reason for changing to more easily edible seeds in this case were the neighbors, i.e., other humans. At the same time, underlaying chain of events was that the neighbors were (perceived) to be annoyed by a plant part that was brought to their property by the birds. While arguably all chains of reasoning result in the person feeding or not feeding birds, the important factor for the main category classification was where the respondents placed the primary reason for the change in their practices.

### Ethical considerations

This research have been performed in accordance with the Declaration of Helsinki. The respondents gave informed consent for participation before entering their responses in the questionnaire. No personal data was asked for. Under the guidelines of the Finnish Advisory Board on Research Integrity, this study did not require institutional ethical review. In open answers, the respondents could provide text that could be considered as identifiable personal or sensitive data (e.g., in explaining how a specific disease might prevent them from easily maintaining a bird-feeding site). These data were handled in line with European Union GDPR regulations, and no answers that had identifiable or sensitive information were published. No birds were harmed during the study.

## Data Availability

The questionnaire data were deposited in Zenodo (10.5281/zenodo.13805256) with the exception of open-answer data that support the findings of this study, which are not openly available as they might contain personal and sensitive information.
